# PDK1 promotes breast cancer progression by enhancing the stability and transcriptional activity of HIF-1α

**DOI:** 10.1016/j.gendis.2023.06.013

**Published:** 2023-07-15

**Authors:** Yu Wei, Dian Zhang, He Shi, Husun Qian, Hongling Chen, Qian Zeng, Fangfang Jin, Yan Ye, Zuli Ou, Minkang Guo, Bianqin Guo, Tingmei Chen

**Affiliations:** a Key Laboratory of Clinical Laboratory Diagnostics (Ministry of Education), College of Laboratory Medicine, Chongqing Medical University, Chongqing 400016, China; b Chongqing Key Laboratory of Translational Research for Cancer Metastasis and Individualized Treatment, Chongqing University Cancer Hospital, Chongqing 400030, China

**Keywords:** Breast cancer, HIF-1α, PDK1, Protein phosphorylation, Transcriptional activity

## Abstract

Pyruvate dehydrogenase kinase 1 (PDK1) phosphorylates the pyruvate dehydrogenase complex, which inhibits its activity. Inhibiting pyruvate dehydrogenase complex inhibits the tricarboxylic acid cycle and the reprogramming of tumor cell metabolism to glycolysis, which plays an important role in tumor progression. This study aims to elucidate how PDK1 promotes breast cancer progression. We found that PDK1 was highly expressed in breast cancer tissues, and PDK1 knockdown reduced the proliferation, migration, and tumorigenicity of breast cancer cells and inhibited the HIF-1α (hypoxia-inducible factor 1α) pathway. Further investigation showed that PDK1 promoted the protein stability of HIF-1α by reducing the level of ubiquitination of HIF-1α. The HIF-1α protein levels were dependent on PDK1 kinase activity. Furthermore, HIF-1α phosphorylation at serine 451 was detected in wild-type breast cancer cells but not in PDK1 knockout breast cancer cells. The phosphorylation of HIF-1α at Ser 451 stabilized its protein levels by inhibiting the interaction of HIF-1α with von Hippel-Lindau and prolyl hydroxylase domain. We also found that PDK1 enhanced HIF-1α transcriptional activity. In summary, PDK1 enhances HIF-1α protein stability by phosphorylating HIF-1α at Ser451 and promotes HIF-1α transcriptional activity by enhancing the binding of HIF-1α to P300. PDK1 and HIF-1α form a positive feedback loop to promote breast cancer progression.

## Introduction

Breast cancer has the highest incidence among cancers and remains the second most fatal cancer in women despite significant advancements in health care management.^1^ Studies have demonstrated that hypoxia and metabolic remodeling are important hallmarks of cancer that promote tumorigenesis.^2^ In breast cancer, intratumorally low oxygen levels (hypoxia) are associated with aggressive tumor behavior, metastasis, and treatment resistance.^3^, ^4^, ^5^ The transcription factor hypoxia-inducible factor 1α (HIF-1α), a major regulator of cellular adaptation to hypoxia, is highly expressed in several cancer types and is associated with poor breast cancer prognosis.^6^ In the hypoxic tumor microenvironment, HIF-1α can adapt tumor cells to the hypoxic environment and induce angiogenesis by activating almost all glycolytic enzyme-related genes and angiogenesis-related genes. However, tumor neovascularization is disorderly and leaky, which further aggravates the adverse tumor microenvironment.^7^ Tumor cells have an active rate of glycolysis, whereas normal cells usually produce ATP through oxidative phosphorylation and only produce ATP through glycolysis when oxygen is scarce.^8^ Tumor cells undergo aerobic glycolysis through metabolic reprogramming even in the presence of sufficient oxygen, thereby lowering the pH of the tumor microenvironment. An acidic and hypoxic tumor microenvironment significantly promotes drug resistance, chemotherapy resistance, metastasis, and tumor cell invasion.^9^

In normoxia, HIF-1α is hydroxylated by the prolyl hydroxylase domain (PHD) protein at the Pro-402 sites and Pro-564 sites, which increases its interaction with the von Hippel-Lindau (VHL) protein, an E3 ubiquitin ligase. The complexes were formed with Cullin 2 (CUL2), Elongin B, and Elongin C and degraded by the proteasome.^10^ However, when there is sufficient oxygen, the enzyme activity of PHD decreases sharply, blocking the breakdown of HIF-1α. In addition, the protein expression of HIF-1α can be altered by post-translational modifications. For instance, polo-like kinase 3 (PLK3) phosphorylates HIF-1α at Ser-576 and Ser-657 sites to promote its degradation.^11^ Protein kinase A (PKA) phosphorylates Thr63 and Ser 692 to increase HIF-1a stability.^12^ These classical and non-classical degradation pathways form the HIF-1α protein regulatory network, which provides important theoretical support for the HIF-1α targeting strategy.

Tumor cells produce energy from glycolysis under oxygen-rich conditions called the Warburg effect.^13^^,^^14^ Although glycolysis is much less efficient than mitochondrial oxidative phosphorylation in producing ATP, tumor cells can obtain several intermediate products from glycolysis to accelerate their anabolism and lower the pH in the microenvironment, which promotes proliferation, invasion, and drug resistance of tumor cells.^15^ Pyruvate dehydrogenase kinase (PDK1) is the key enzyme of the glycolysis pathway, mainly distributed in the mitochondrial matrix, and includes four subtypes: PDK1, PDK2, PDK3, and PDK4.^16^ PDKs inhibit the activity of pyruvate dehydrogenase complex by phosphorylating the E1a subunit at sites 293, 300, and 232, thereby inhibiting the tricarboxylic acid cycle and promoting glycolysis, an important target of the Warburg effect.^8^^,^^17^ HIF-1α-mediated activation of PDK1 transcription is a gatekeeper of oxidative phosphorylation in hypoxia.^18^ The function of PDK1, an oncogene, has been reported in vascular growth, cell proliferation, and migration.^19^^,^^20^ In addition, studies have reported that PDK1 is involved in breast cancer progression, chemotherapy resistance, and distant metastasis, although controversies remain.^21^^,^^22^

To fully elucidate the role of PDK1 in breast cancer, we first investigated whether PDK1 can promote the progression of breast cancer. The results showed that PDK1 promoted the HIF-1α pathway in the mouse model of burdened subcutaneous tumors. We also investigated the mechanism through which PDK1 regulates the HIF-1α protein level. The present study clarified whether PDK1 affects the protein stability and transcriptional activity of HIF-1α. It was experimentally demonstrated that HIF-1α under hypoxic conditions induced PDK1, a downstream target gene of HIF-1α, which promotes protein stability and transcriptional activity of HIF-1 α, forming a positive feedback loop that promotes breast cancer progression. We identified a novel role and regulatory mechanism of the PDK1/HIF-1α positive feedback loop in breast cancer, which provides a promising strategy for breast cancer treatment.

## Materials and methods

### Cell lines

All cell lines were obtained from the American Type Culture Collection (ATCC; Rockville, MD, USA). BT-549 cell lines were cultured in RPMI 1640 medium (Basal Medium, CHINA) containing 20% fetal bovine serum (FBS; Gibco, Grand Island, NY, USA) at 37 °C in 5% CO_2_. HCC1806 cell lines were cultured in RPMI 1640 medium (Basal Medium, CHINA) with 10% fetal bovine serum (FBS; Gibco, Grand Island, NY, USA) at 37 °C in 5% CO_2_. MCF-7, HEK-293T, and HUVEC cell lines were cultured in DMEM medium (Basal Medium, CHINA) with 10% fetal bovine serum (FBS; Gibco, Grand Island, NY, USA) at 37 °C in 5% CO_2_.

### RNA extraction and quantitative real-time PCR

For gene expression analysis, total RNA was extracted with Tizol reagent (Takara, Japan, 9109) and reverse-transcribed into cDNA using the PrimeScript RT reagent kit (Takara, Japan, RR037A). The mRNA levels of the indicated genes were detected by qRT-PCR using SYBR Premix Ex Taq II (TaKaRa, Japan, RR820A) by CFX96 Touch Real-Time PCR Detection System (Bio-Rad, USA). The relevant quantitative PCR primer sequences are shown in Table 1.

**Table 1:** Primers of QRT-PCR.

	Sequence (5′→3′)	
ANGPT1	Forward: AGCGCCGAAGTCCAGAAAAC	Reverse: TACTCTCACGACAGTTGCCAT
EGF	Forward: TGGATGTGCTTGATAAGCGG	Reverse: ACCATGTCCTTTCCAGTGTGT
FLT1	Forward:TTTGCCTGAAATGGTGAGTAAGG	Reverse: TGGTTTGCTTGAGCTGTGTTC
SDF1	Forward:ATTCTCAACACTCCAAACTGTGC	Reverse: ACTTTAGCTTCGGGTCAATGC
P21	Forward: TGTCCGTCAGAACCCATGC	Reverse: AAAGTCGAAGTTCCATCGCTC
PFKL	Forward: GCTGGGCGGCACTATCATT	Reverse: TCAGGTGCGAGTAGGTCCG
ENO1	Forward: AAAGCTGGTGCCGTTGAGAA	Reverse:GGTTGTGGTAAACCTCTGCTC
REDD1	Forward: TGAGGATGAACACTTGTGTGC	Reverse: CCAACTGGCTAGGCATCAGC
NIX	Forward:TTGGATGCACAACATGAATCAGG	Reverse:TCTTCTGACTGAGAGCTATGGTC
TIMP1	Forward:CTTCTGCAATTCCGACCTCGT	Reverse:ACGCTGGTATAAGGTGGTCTG
VEGFA	Forward:AGGGCAGAATCATCACGAAGT	Reverse:AGGGTCTCGATTGGATGGCA

### Protein extraction and Western blotting

After three washes with ice-cold PBS, cells were lysed with RIPA lysis buffer (Beyotime, P0013B) containing proteinase inhibitor cocktail (APExBIO, K1007) and phosphatase inhibitor cocktail (APExBIO, K1015). The lysates were incubated on ice for 30 min and then centrifuged at 14,500 *g* for 20 min. Then, the protein concentration was quantified using the BCA kit (Takara, Tokyo, Japan) and denatured with 5 × SDS sample buffer at 100 °C for 10 min. Proteins were separated by 6%–12% SDS-PAGE and transferred onto 0.45-μm PVDF membranes (Millipore Corporation, Billerica, MA, USA). The membranes were blocked with 5% non-fat milk in TBS buffer with 0.1% Tween 20 for 1 h at room temperature. After incubating the primary antibody overnight at 4 °C, the membrane was washed thrice with TBS-T and incubated with the secondary antibody (goat anti-rabbit HRP or goat anti-mouse HRP, 1:10,000, Biosharp) at room temperature for 1 h. The proteins of interest were visualized after exposing the membranes to an ECL substrate solution (Millipore, WBKLS0100). The specific primary antibodies used in Western blotting analysis were as follows: anti-PDK1 (1:1000, Abcam, ab207450), anti-α-Tubulin (1:1000, Proteintech, 66031-1-Ig), anti-DYKDDDDK tag (1:8000, Proteintech, 20543-1-AP), anti-PDHE1A (1:5000, Proteintech, 180681-1-AP), anti-Phospho-PDHE1A(S232) (1:1000, abcpta, ap3758A), anti-P300 (1:200, Santa Cruze, sc-48343), anti-Ub (1:200, Santa Cruze, sc-8017), and anti–HIF–1α (1:1000, Proteintech, 20960-1-AP).

### Immunofluorescence (IF)

A thousand cells were seeded in a confocal dish for 24 h. Subsequently, the cells were washed thrice with ice-cold PBS and fixed with 4% paraformaldehyde for 20 min. Then the cells were permeabilized with 0.1% Triton X-100 for 10 min and blocked with 0.5% goat serum for 1 h. The specific antibodies against PDK1 (Santa Cruze, sc-515,944, 1:50) and HIF-1a (Proteintech, 20960-1-AP, 1:100) were incubated with cells overnight at 4 °C. Then, the cells were incubated with myc-labeled or FITC-labeled secondary antibodies in the dark at room temperature for 1 h. The cell nuclei were stained with DAPI (Boster, AR1176) for 15 min. Immunofluorescence staining was detected with the confocal microscope.

### Immunohistochemistry (IHC)

Paraffin sections of 4-uM tumor tissues were roasted on Slide Warmer at 60 °C for 1 h, deparaffinized with xylene, and rehydrated with gradient ethanol. The slides were heated with citric acid buffer (pH 6.0) at 95 °C for 20 min for antigen retrieval. The slides were then treated with 3% H2O2 for 10 min to block the endogenous peroxidase reactivity. Subsequently, 5% goat serum was used to block non-specific proteins. The sections were separately incubated with primary antibodies against CD31 (Servicebio, GB11063-1, 1:200), ki67 (Servicebio, GB111141, 1:200), and HIF-1a (Proteintech, 20960-1-AP,1:100). Subsequently, the slides were washed and then incubated with secondary antibodies at room temperature for 30 min. The color was developed with DAB for 1 min and hematoxylin for 1 min. Images were captured using microscopy (Nikon ECLIPSE Ti-s, Japan).

### Luciferase assay

BT-549 cells were seeded in a 6-well plate and transfected with 500 ng pGMLR-TK and 500 ng pGMHIF-1-Lu plasmids. The former served as a control plasmid, while the latter contained a 3XHRE, and both were conducted for 36 h. The cells were treated with CoCl_2_ for 6 h, and MG132 for 4 h. Cell lysates were analyzed by the Luc-Pair™ Duo-Luciferase HS Assay Kit (GeneCopoeia) according to the instructions. The firefly luciferase luminescence data were normalized using that of the Renilla luciferase luminescence data.

### Cell proliferation assay

A total of 1000 cells were seeded in 96-well plates and incubated at 37 °C in a humidified atmosphere containing 5% CO_2_. The cells were subjected to the Cell Counting Kits-8 (CCK8) assay and the results were analyzed by measuring the absorbance every 12 h until 48 h using a spectrophotometer.

### Colony formation

A total of 1000 cells were seeded in a 60-mm dish and then cultured for two weeks. After staining with crystal purple, the number of colonies was counted using ImageJ.

### Co-immunoprecipitation (Co-IP)

The magnetic beads were cleaned thrice with PBST and incubated with specific antibodies or IgG overnight at 4 °C. The cells were collected in a 1.5-mL EP tube, lysed in IP lysis buffer with proteinase inhibitor cocktail (APExBIO, K1007) and phosphatase inhibitor cocktail (APExBIO, K1015). Lysates were sonicated and incubated on ice for 30 min. The lysates were centrifuged and incubated with antibody-coated magnetic beads for 1 h at room temperature for Co-IP. Then, the magnetic beads were washed thrice with PBST, resuspended in a loading buffer, boiled, and subjected to SDS–PAGE.

### Chromatin immunoprecipitation assay (ChIP)

Chromatin immunoprecipitation analysis was performed using the SimpleChIP® Enzymatic Chromatin IP Kit (Cell Signaling Technology, #9003). Briefly, the cells were cross-linked with 1% formaldehyde and then blocked with glycine. Subsequently, the cells were washed thrice with pre-chilled PBS and treated with micrococcal nuclease. Then, the pellet was suspended in a ChIP buffer and sonicated. The chromatin was then incubated with HIF-1α antibody to enrich DNA, and qRT-PCR was used to quantify the enriched DNA with ChIP primers.

### Tubule formation assay

HUVEC cells were used for the *in vitro* tube formation assay. Reduced-growth factor Matrigel (Corning, Cat# 354,234) was thawed on ice. Then, 50 μL of Matrigel was added to 96-well plates for polymerization and incubated for 30 min in a 37 °C incubator. A total of 1 × 104 HUVEC cells cultured with the supernatants of the indicated cancer cells were seeded on top of the Matrigel in each well. After incubation at 37 °C for 6 h, images of tube formation in each well were captured using microscopy (Nikon ECLIPSE Ti-s, Japan) and analyzed using ImageJ software.

### Generation of PDK1-sh cell lines

We designed two sgRNA oligonucleotides for PDK1 to silence the expression of PDK1 in BT-549 cells and tested their effects on gene silencing. The sgRNA sequences for the PDK1 gene were 5′- GCAAGTACAATGGCACTGCG -3′ and 5′- AAAGTTTGGTGATACTATAG -3′. As single-cell clone cells grew, multiple clones were selected to extract the protein for Western blot analysis.

### Generation of PDK1-KO cell lines

To stably knock out PDK1 gene in BT-549 cells, we designed two sgRNA oligonucleotides for PDK1 and tested their effects on gene silencing. The sgRNA sequence for PDK1 gene were 5′- GCAAGTACAATGGCACTGCG -3′ and 5′- AAAGTTTGGTGATACTATAG -3′. When single-cell clone cells grew, several clones were picked to extract the protein for Western blot analysis.

### Tumorigenicity in nude mice

The tumorigenic ability of the cells was evaluated by subcutaneous inoculation of 1 × 106 MCF7 cells into 4-week-old female mice (*n* = 5, per group). The tumor volume of nude mice was measured every three days (tumor volume = 0.5 × length × width^2^). The animals were sacrificed three weeks after inoculation. The Institutional Animal Care and Ethical Committee of Chongqing Medical University approved all animal experiments.

## Results

### High PDK1 expression in breast cancer cells was closely related to tumor progress

**Figure 1:**
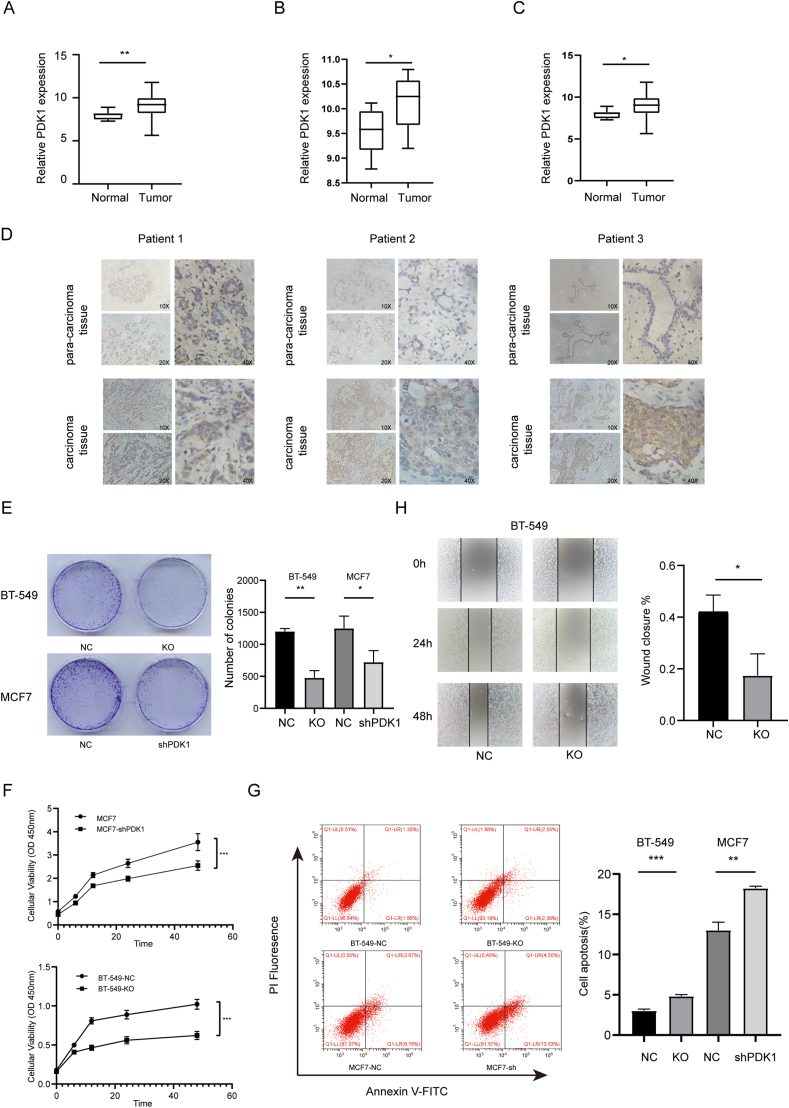
PDK1 was up-regulated in breast cancer and promoted the proliferation and migration, and inhibited apoptosis of breast cancer cells. **(A**–**C)** The PDK1 mRNA level was higher in breast cancer patients than in healthy people. The PDK1 mRNA expression data were retrieved from the following Gene Expression Omnibus datasets: GSE65194, GSE33447, and GSE45827. **(D)** Representative IHC staining images of PDK1 in breast cancer tissues and para-carcinoma tissue. **(E)** Left panel: colony formation experiments showing the clone numbers of BT-549 cells and MCF7 cells. Right panel: quantification of colonies in experiments performed in three replicates under the same condition. **(F)** CCK8 assays were conducted to determine the viability of breast cancer cells following PDK1 knockdown or knockout. **(G)** The apoptotic rates (LR + UR) of breast cancer cells were detected by flow cytometry (Q2 + Q3). LR, early apoptotic cells; UR, terminal apoptotic cells. Right panel: quantification of flow cytometric results from three replicates under the same condition. **(H)** Left panel: representative images of wound-scratch assay of BT-549-NC and BT-549-KO cell lines. Right panel: quantification of wound-scratch assay results from three replicates under the same condition.

Using CancerMinea, a literature-based resource, we summarized that PDK1 might behave as an oncogene in many cancers, including breast cancer (Fig. S1A). We analyzed the expression of PDK1 in the GSE65194, GSE33447, and GSE45827 datasets to elucidate the cancerogenic role of PDK1 in breast cancer. Compared with normal people, breast cancer patients had higher expression of PDK1 (Fig. 1A–C). Also, the immunohistochemical analysis indicated that PDK1 expression was higher in the human breast cancer tissues than in the adjacent normal tissues (*n* = 19) (Fig. 1D; Fig. S1B). Cancer cells often exhibit malignant characteristics, such as increased proliferation, migration, and anti-apoptotic ability. To determine whether the high expression of PDK1 affected the malignant behavior of breast cancer cells, we generated the PDK1 stable knockdown cell line MCF7-sh and PDK1 stable knockout cell line BT-549-KO through sh-RNA interference and sgRNA knockout (Fig. S1C). The CCK8 and colony formation assays were then to determine the proliferation of cancer cells. It was observed that PDK1 knockdown or knockout suppressed cell proliferation significantly (Fig. 1E, F). Meanwhile, flow cytometry indicated that PDK1 markedly inhibited the apoptosis of tumor cells (Fig. 1G). In addition, we performed a scratch assay to explore the migratory capacity of BT-549. Compared with the control cells, the PDK1-KO cells exhibited significantly delayed migration ability (Fig. 1H). In addition, we treated T-47D and MDA-MB-468 cells with DCA (Dichloroacetate, a pyruvate dehydrogenase kinase inhibitor that targets the pyruvate binding site of PDKs and inhibits their kinase activity) and observed trend changes (Fig. S1D–G). Results showed that PDK1 was highly expressed in breast cancer. After PDK1 down-regulation, the proliferation, migration, and anti-apoptotic properties of breast cancer cells were markedly inhibited.

### Depletion of PDK1 in breast cancer cells displayed tumor growth retardation and inactivation of the HIF-1α signaling pathway

**Figure 2:**
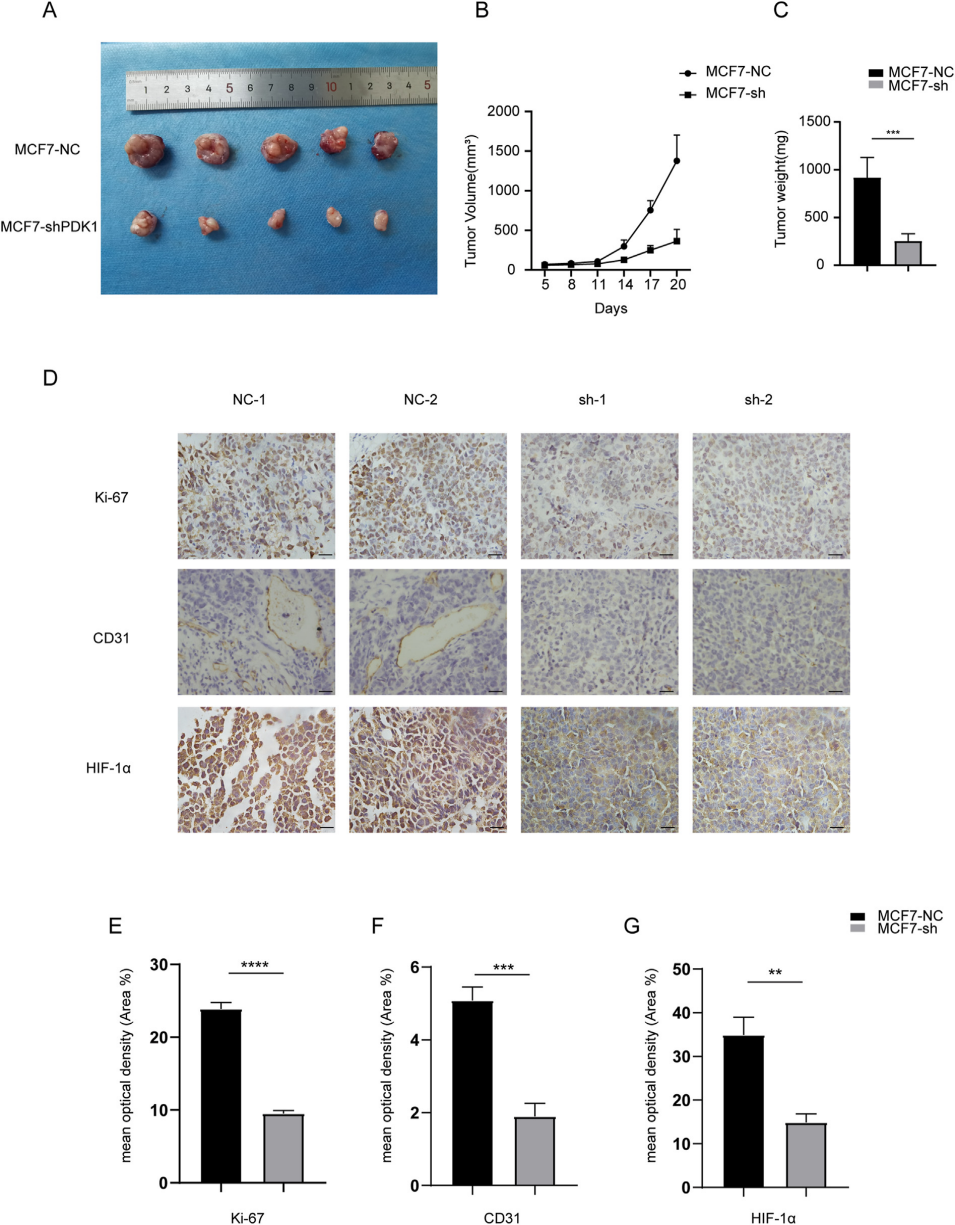
Depletion of PDK1 significantly inhibited tumor growth and caused HIF-1α inactivation **(A**–**C)** Representative tumor size, tumor volume, and tumor weight for each group of xenograft mice (5 mice in each group) are shown (^∗∗∗^*P* < 0.001). Mice were sacrificed and tumor tissues were collected and photographed. The tumor volume and tumor weight were calculated at the end of the experiment **(D)** Representative IHC staining images of Ki-67, CD31, and HIF-1α in MCF7-NC and MCF7-sh-PDK1 (scale bars, 50 μm) **(E**–**G)** Quantification of Ki-67, CD31, and HIF-1α in MCF7-NC and MCF7-sh-PDK1 (^∗∗^*P* < 0.01, ^∗∗∗^*P* < 0.001).

Having established that PDK1 promoted the proliferation and migration of breast cancer cells *in vitro*, we further investigated whether PDK1 affects tumor growth *in vivo*. We subcutaneously injected Con-shRNA or PDK1-shRNA MCF7 cells into BALB/C nude mice and sacrificed them three weeks later. It was observed that tumors derived from PDK1-shRNA MCF7 cells had a significantly lower volume and weight than control tumors (Fig. 2A–C). Immunohistochemical staining showed that the expression of Ki-67, a marker used to label cells in the proliferative cycle, was significantly higher in the control group than in the PDK1-shRNA group (Fig. 2D, E). Treatment of MCF7 cells with DCA also considerably reduced tumor size and mass (Fig. S2A–C). Interestingly, we observed that the tumor surface of PDK1-shRNA MCF7 cells was pale and had fewer blood vessels (Fig. 2A). Moreover, immunohistochemical staining of tumors revealed that PDK1-shRNA tumors lower CD31 expression, a marker used to evaluate tumor angiogenesis (Fig. 2D, F). Since the HIF-1 signaling pathway is closely linked to angiogenesis, we explored whether the transcription of PDK1 induced by HIF-1α could in turn affect the HIF-1α expression levels.^23^^,^^24^ As expected, less HIF-1α was detected in the PDK1-sh group than in the control group (Fig. 2D, G). Meanwhile, the correlation analyses via the GEPIA platform from TCGA data revealed that PDK1 was positively correlated with HIF-1α and its downstream genes VEGFA and ENO1 (Fig. S2D), suggesting that PDK1 has a potential regulatory mechanism for HIF-1α.

### PDK1 promoted HIF-1α protein stability

**Figure 3:**
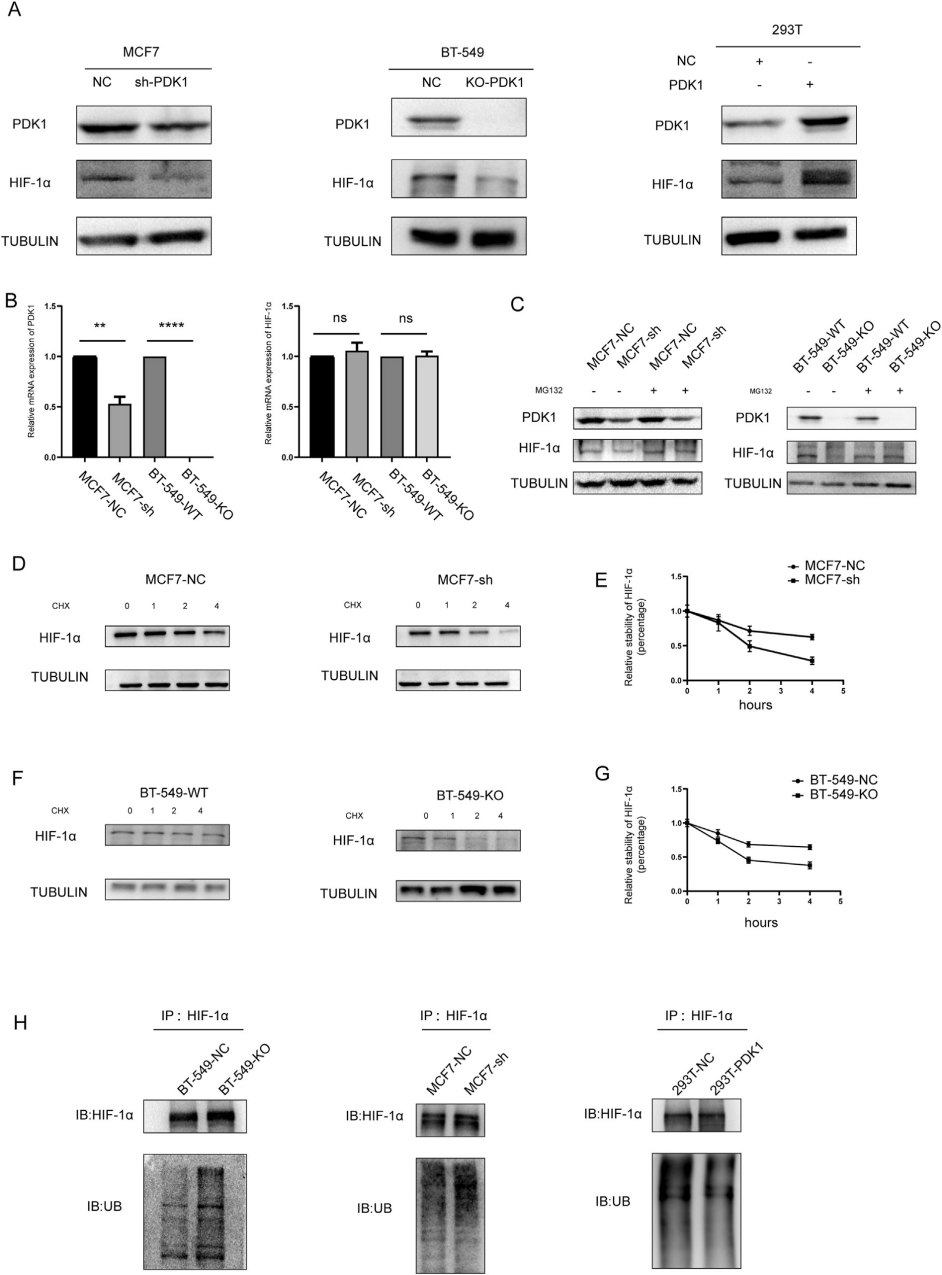
PDK1 promoted HIF-1α protein stability. **(A)** The protein expression of HIF-1α was detected by Western blotting and normalized to the expression of TUBULIN. HEK-293T cells were transfected with empty vector (NC) or PDK1 overexpression plasmid. All cells were treated with CoCl_2_ for 6 h to simulate hypoxia before experimentation. **(B)** The mRNA levels of HIF-1α and PDK1 in indicated cells were determined by qRT-PCR. Gene expression was normalized to TUBULIN as a reference gene (^∗∗^*P* < 0.01, ^∗∗∗∗^*P* < 0.0001). All cells were treated with CoCl_2_ for 6 h before experimentation. **(C)** The expression level of HIF-1α in the indicated cells was detected by Western blotting and normalized to TUBULIN as the reference gene. All cells were treated with CoCl_2_ for 6 h and MG132 for 4 h before experimentation. **(D, F)** The expression level of HIF-1α in MCF7 and BT-549 cells was detected by Western blotting and normalized to TUBULIN. All cells were treated with CoCl_2_ for 6 h and 25 ug/mL cycloheximide (CHX) for indicated time points (0, 1, 2, 4 h) before experimentation. **(E, G)** Quantification of protein levels of HIF-1α. In each group, the protein level of HIF-1α at 0 h was recorded as 100%. **(H)** Immunoprecipitation assays were conducted to measure the ubiquitination levels of HIF-1α in the indicated cells. The cell lysates were subjected to immunoprecipitation assay using HIF-1α antibody and detected by Western blot using the indicated antibodies. All cells were treated with CoCl_2_ for 6 h to simulate hypoxia before experimentation.

The mechanism through which PDK1 promotes the expression of HIF-1α was also explored. First, the expression of HIF-1α in breast cancer cells was characterized using Western blotting. Knockdown of PDK1 by sh-RNA in MCF7 cells and knockout of PDK1 by CRISPR in BT-549 cells decreased the expression of HIF-1α (Fig. 3A). Conversely, the overexpression of PDK1 significantly increased the expression of HIF-1α in 293T cells (Fig. 3A). Similarly, after treating T-47D and MDA-MB-468 cells with DCA to inhibit the activity of PDKs, HIF-1α protein levels were significantly reduced (Fig. S2E). We quantified the mRNA level of HIF-1α in breast cancer cells using qRT-PCR to clarify whether the regulatory mode of PDK1 on HIF-1α belongs to pre-transcriptional regulation or post-transcriptional regulation. The results showed that the mRNA level of HIF-1α did not change significantly between breast cancer cell groups, indicating that the regulation of HIF-1α by PDK1 may be transcriptionally independent (Fig. 3B; Fig. S2F). Therefore, it was hypothesized that PDK1 promotes HIF-1α expression by inhibiting its degradation. In BT-549 and MCF7 cells with PDK1 depletion, the protein level of HIF-1α was hardly detected. However, upon treatment with MG132, an inhibitor was used to inhibit proteasome activity, and the HIF-1α protein could accumulate rapidly (Fig. 3C). Cells were treated with cycloheximide, an inhibitor of protein synthesis in eukaryotes, to assess the stability of HIF-1α. Consistent with previous findings, it was observed that the existence of PDK1 could enhance HIF-1α protein stability in MCF7 and BT-549 cells (Fig. 3D, E). The classical HIF-1α degradation pathway is through the PHD–HIF–1α-VHL axis to increase its ubiquitin levels and be degraded by the proteasome. To assess whether the effect of PDK1 on the stability of the HIF-1α protein was due to the regulation of its ubiquitination, we detect the ubiquitination level of endogenous HIF-1α by co-immunoprecipitation. The results showed that the ubiquitin level of HIF-1α in cell lines with high expression of PDK1 was significantly decreased, that is, PDK1 reduced the ubiquitination of HIF-1α, thereby stabilizing its protein level (Fig. 3F).

### PDK1 interacted with HIF-1α through its C-terminal HATPase domain and promoted HIF-1α phosphorylation at Ser 451

**Figure 4:**
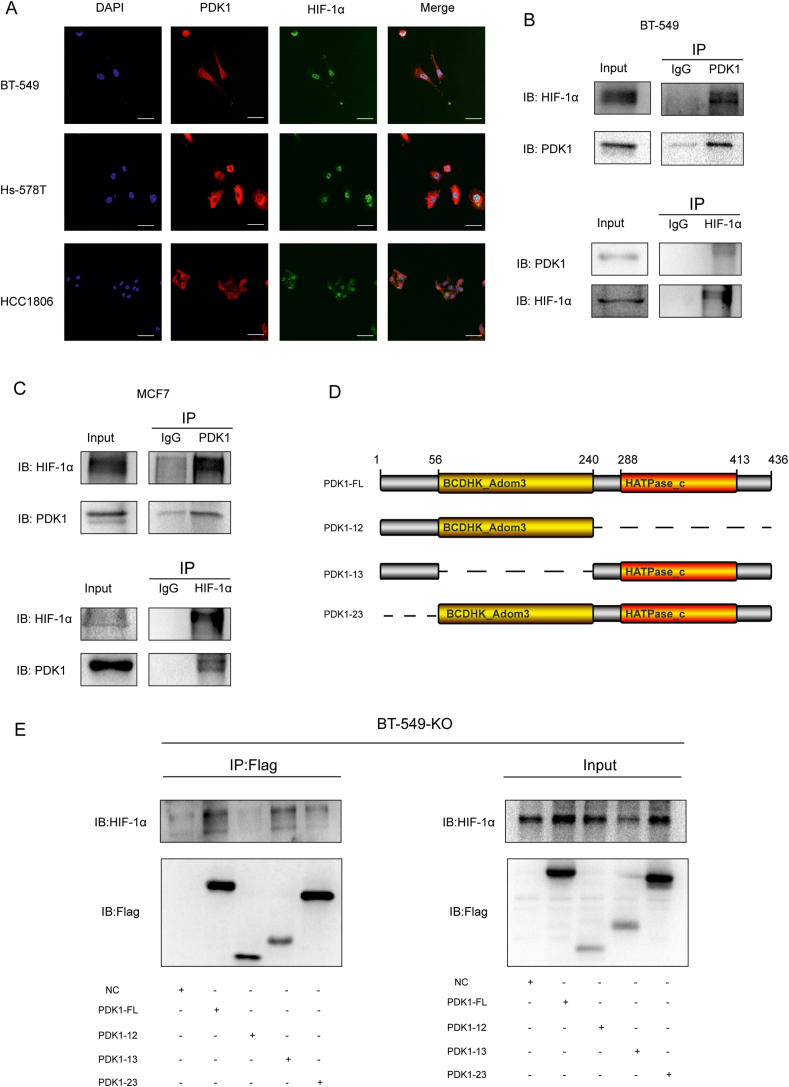
PDK1 interacted with HIF-1α through its C-terminal HATPase domain. **(A)** Immunofluorescent detection of PDK1 (red) and HIF-1α (green) in the indicated cells following treatment with CoCl_2_ for 6 h (Scale bars, 50 μm). **(B)** Immunoprecipitation was performed to detect the interaction between PDK1 and HIF-1α in BT-549-NC and BT-549-KO cells pretreated with CoCl_2_ for 6 h. **(C)** Immunoprecipitation assay results showing the interaction between PDK1 and HIF-1α in MCF7-NC and MCF7-sh-PDK1 cells pretreated with CoCl_2_ for 6 h. **(D)** A schematic illustration of the functional domains of PDK1 and truncated constructs. **(E)** Vector or flag-tagged different PDK1 fragment-overexpressing in BT-549-KO cells were immunoprecipitated with anti-FLAG Magnetic Beads and immunoblotted. The interaction between HIF-1α and different truncated PDK1 was detected.

**Figure 5:**
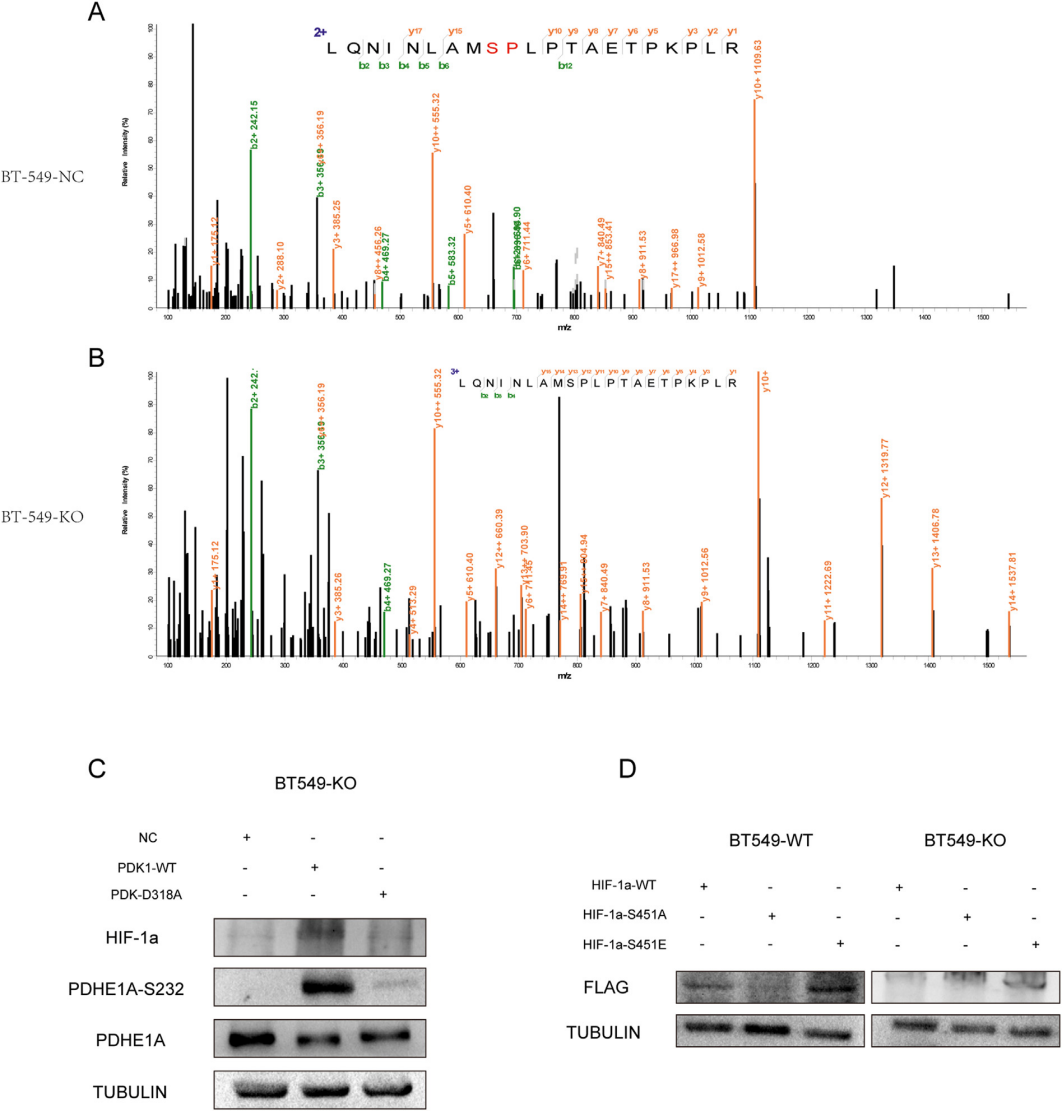
PDK1 phosphorylated HIF-1α at Ser 451 to promote its stability. **(A, B)** Mass spectrometry analysis identified S451 of HIF-1α as the site for PDK1-induced phosphorylation. Phosphorylated HIF-1α was digested with trypsin and then analyzed by LC-MS/MS. The LC/MS mass spectrum of a phosphorylated peptide is shown for peptide HIF-1α 443–463 containing phosphorylated Ser451. **(C)** Immunoblotting was performed to explore whether the expression of HIF-1α protein was affected by PDK1 kinase activity. BT-549-KO cells were transfected with empty vector (NC) plasmid, wild-type PDK1(PDK1-WT) plasmid, or PDK1-D318A mutant plasmid, and treated with CoCl_2_ for 6 h before experimentation. **(D)** Flag–HIF–1α levels in the indicated cells were detected by Western blotting and results were normalized to TUBULIN as the reference gene. BT-549-WT cells and BT-549-KO cells were transfected with Flag-tagged HIF-1α wild-type (HIF-1α-WT) plasmid, HIF-1α-S451A mutant plasmid, or HIF-1α-S451E mutant plasmid, and treated with CoCl_2_ for 6 h before experimentation.

The mechanisms and functional implications of the PDK1-dependent regulation of HIF-1α were further elucidated. HIF-1α protein stability is regulated by the classical PHD2- HIF-1α-VHL proteasome pathway and various HIF-1α non-classical degradation pathways. CDK1 and PKA, two phosphokinases, inhibited HIF-1α degradation by phosphorylating HIF-1α protein at Ser 668, Thr63, and Ser 692.^12^^,^^25^ It was hypothesized PDK1, as a kinase, might regulate the stability of HIF-1α protein through a similar pathway. First, we investigated the interaction between PDK1 and HIF-1α. Using immunofluorescence assays, we found the strong co-localization of PDK1 and HIF-1α in BT-549, HS-578T, and HCCT-1886 cells (Fig. 4A). This interaction was further corroborated using immunoprecipitation in BT-549 and MCF7 cells (Fig. 4B, C). A PDK1 expression vector was transiently transfected into 293T cells with a HIF-1α expression plasmid to explore the exogenous interaction between PDK1 and HIF-1α. Therefore, the data suggested that exogenous and endogenous PDK1 could interact with HIF-1α (Fig. S3A). We constructed truncated mutations of PDK1 according to the structure of PDK1, including the mitochondrial localization sequence (1–56), the BCDHK_Adom3 domain (56–220), and the HATPase domain (220–436) to verify the specific domain of PDK1 that interacted with HIF-1α (Fig. 4D). The lack of interaction of Flag-PDK1 with HIF-1α confirmed the specificity of the binding, which was based on the HATPase domain (Fig. 4E). Given the kinase activity of PDK1, we verified whether HIF-1α could be phosphorylated as a substrate of PDK1. First, we explored whether the HIF-1α protein levels depended on PDK1 kinase activity. The D318 of PDK1 is the ATP-binding site, and the D318A mutated PDK1, which inhibits ATP binding to PDK1, was constructed to inhibit the kinase activity of PDK1. Since PDK1 is the sole kinase in the PDK family that can phosphorylate the PDHE1α site at Ser 232,^17^ we determined the enzymatic activity of PDK1 by measuring the phosphorylation level of Ser232 of PDHE1α. As expected, HIF-1α expression was rescued after transferring PDK1-WT plasmids into PDK1 knockout BT-549 cells, while the transfected PDK1-D318A mutant plasmid could not (Fig. 5C). These results demonstrated that the HIF-1α protein levels were dependent on PDK1 kinase activity. We performed immunoprecipitation and liquid chromatography-tandem mass spectrometry (LC-MS/MS) analyses of HIF-1α in BT-549-NC and BT-549-KO cells to explore the phosphorylation site of PDK1 on HIF-1α. We found phosphorylation at Ser 451 of HIF-1α in BT-549-NC that was not identified in BT-549-KO cells (Fig. 5A, B). The phosphorylation of HIF-1α at Ser451 plays an important role in maintaining the stability of the HIF-1α protein and interrupts the interaction of HIF-1α with PHD and pVHL.^26^ BT-549-NC and BT-549-KO cells were transfected with the flag-tagged wild-type HIF-1α plasmid, the S451A mutant (the dephosphorylation-mimetic) HIF-1α plasmid or S451E mutant (the phosphorylation-mimetic) HIF-1α plasmid to verify whether phosphorylation of HIF-1α at S451 affects its protein stability. In BT-549-NC cells, the wild-type HIF-1α protein level was significantly higher than that of the S451A mutant type, and the S451E mutant type dramatically rescued HIF-1α protein levels (Fig. 5C). Similarly, in BT-549-KO cells, there was no significant difference in protein levels between wild-type HIF-1α and the S451A mutant type, and the S451E mutant type also rescued the HIF-1α protein levels (Fig. 5C). These results highlighted that PDK1 phosphorylated HIF-1α at S451 and promoted its protein stability.

### PDK1 enhanced the transcriptional activity of HIF-1α

**Figure 6:**
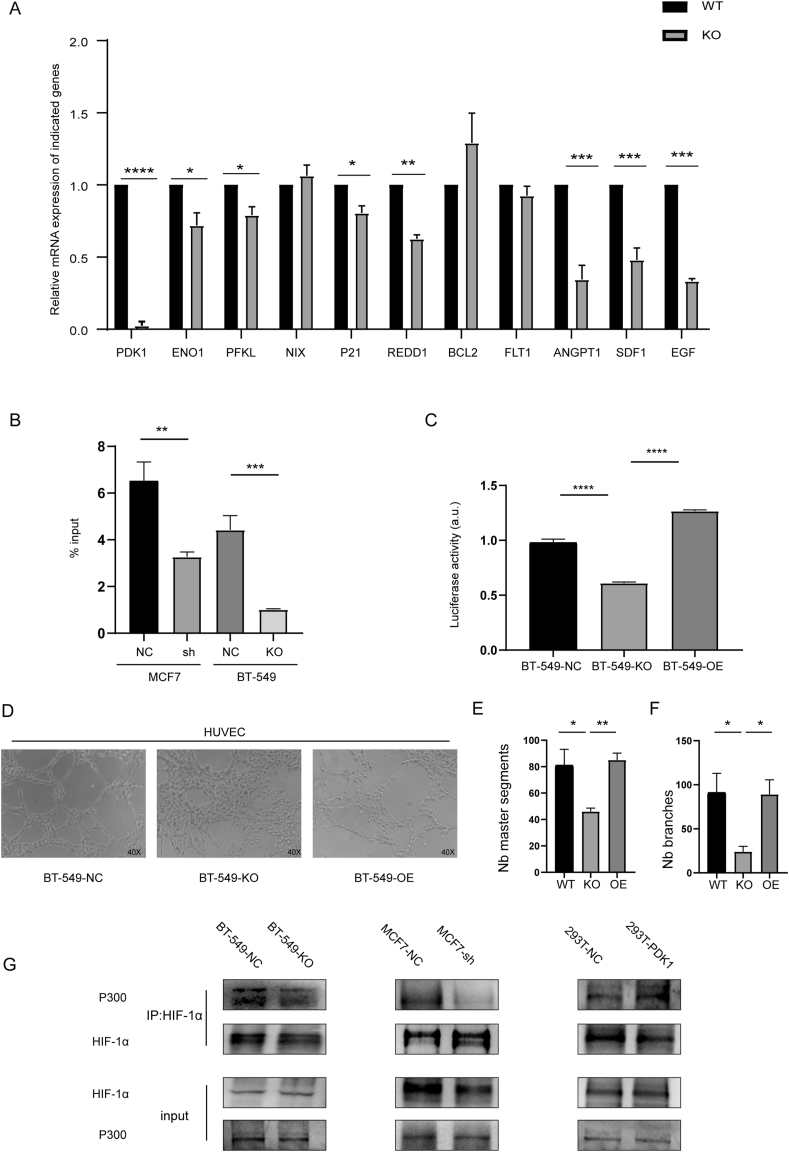
PDK1 enhanced the transcriptional activity of HIF-1α. **(A)** The mRNA expression of HIF-1α in BT-549-NC and BT-549-KO cells was measured with Q-PCR. **(B)** ChIP assays were performed with IgG or anti–HIF–1α antibodies in BT-549-NC cells and BT-549-KO cells. Once the ChIP was performed, qPCR reactions were set up in triplicate for each ChIP sample. **(C)** BT-549-NC cells, BT-549-KO cells, and BT-549-OE cells were co-transfected with PGMHIF-1-Lu and pRL-TK-Renilla, and treated with CoCl_2_ for 6 h and MG132 for 4 h before experimentation. The ratio of firefly/Renilla luciferase was determined (^∗∗∗∗^*P* < 0.0001). **(D)** Representative images showing the formation of HUVEC tubes following incubation with supernatants derived from BT-549-NC cells, BT-549-KO cells, and BT-549-OE cells treated with CoCl_2_ for 6 h; the number of master segments **(E)** and the number of branches **(F)** were calculated (^∗^*P* < 0.05, ^∗∗^*P* < 0.01). **(G)** Immunoprecipitation assays were performed to detect the interaction between HIF-1α and P300 in the indicated cells. 293T cells were transfected with empty vector (NC) plasmid or PDK1 expression plasmid and treated with CoCl_2_ for 6 h.

Considering that PDK1 has nuclear localization (Fig. 4A), we determined whether hypoxia could alter the nucleoplasmic distribution of PDK1. The immunofluorescence experiments showed that hypoxia promoted PDK1 nucleation (Fig. S3F). Nuclear translocation of metabolic enzymes usually regulates cellular transcriptional activity. We also investigated whether PDK1 could promote the HIF-1α transcriptional activity. qRT-PCR was performed on the downstream genes of HIF-1α in BT-549-NC and BT-549-KO cells to observe the effect of PDK1 on the transcriptional activity of HIF-1α. As expected, the mRNA levels of genes related to the glycolysis pathway and proliferation apoptosis pathway, and especially those related to the angiogenesis pathway, were significantly decreased in BT-549-KO (Fig. 6A). Similar results and trends were observed in MCF7-NC and MCF7-sh cells, as well as in DCA-treated T-47D and MDA-MB-468 cells (Fig. S3B–D). Chip-qPCR assays showed that PDK1 enhanced HIF-1α occupancy on ANGPT1 HRE (hypoxia response element) in BT-549 and MCF7 cells (Fig. 6B). We also performed a luciferase assay to observe the effect of PDK1 on HIF-1α transcriptional activity. We constructed a BT-549-OE cell line stably expressing PDK1 in BT-549-KO (Fig. S3C). PGMHIF-1-Lu plasmids, consisting of a triple hypoxic reaction element (3XHRE) and pRL-TK plasmids, were co-transfected into BT-549-NC, BT-549-KO, and BT-549-OE cells, the cells were treated with MG132 before the experiment to make the expression of HIF-1α equal (Fig. S3E). The luciferase activity in BT-549-KO cells was significantly lower than in BT-549-NC cells. The luciferase activity of HIF-1α was restored following the restoration of PDK1 expression (Fig. 6C). According to the previous results, the effect of PDK1 on HIF-1α downstream molecules was mainly focused on the angiogenic pathway. We then tested the effect of PDK1 on HIF-1α transcriptional activity through angiogenesis. HUVECs cells were cultured in different supernatants to assess the angiogenesis capacity, and PDK1 knockout was found to significantly reduce the angiogenesis capacity of HUVEC (Fig. 6D–F). We further explored the mechanism of PDK1 that promotes HIF-1α transcriptional activity. The histone acetyltransferase p300, a histone acetyltransferase, can form a complex with HIF-1α and regulate transcription via chromatin remodeling.^27^ We also determined whether PDK1 regulated the interaction between HIF-1α and p300 through co-immunoprecipitation. Notably, inhibition of PDK1 weakened the interaction between HIF-1α and p300, while the interaction between the two was significantly enhanced after the expression of PDK1 in HEK-293T (Fig. 6G). P DK1 enhanced HIF-1α transcriptional activity by recruiting p300 to facilitate downstream gene expression.

## Discussion

As the gatekeeper of the Warburg effect, PDK1 modulates metabolic reprogramming by regulating pyruvate dehydrogenase complex activity and is a promising therapeutic target in many cancers. Previous studies highlighted that PDK1 promoted tumor development through several signal transduction mechanisms involved in senescence, metastatic tropism, and tumor maintenance.^14^^,^^28^ We found that high expression of PDK1 in breast cancer cells significantly promoted cell proliferation and migration and inhibited cell apoptosis. In addition, a subcutaneous tumor model in nude mice was established, and the tumorigenesis function of PDK1 was verified. The results showed that inhibiting PDK1 decreased HIF-1α expression and reduced neovascularization. The HIF-1α pathway plays a major role in tumors and promotes tumor progression.^29^^,^^30^ Since tissue hypoxia is a normal phenomenon in tumors, future studies should investigate whether PDK1 and HIF-1α form a positive feedback loop that regulates tumor progression.

We also found that PDK1 protected HIF-1α from proteasome degradation and promoted protein stability by reducing its ubiquitination level. However, the underlying mechanism is still unknown. The stability of HIF-1α could be regulated by various post-translational modifications, including phosphorylation, deacetylation, SOMOylation, and ubiquitination.^31^ We postulated that PDK1, as a phosphokinase, maintained protein stability by phosphorylating HIF-1α. Since HIF-1α is a substrate for PDK1, we presupposed that HIF-1α interacts with PDK1, and the interaction between PDK1 and HIF-1α was confirmed with immunofluorescence and immunoprecipitation. PDK1 binds to HIF-1α via its C-terminal HATPase domain. In addition, we applied the PDK1-D318A mutation, which could inactivate PDK1 kinase activity, and observed the expression of HIF-1α in PDK1-KO BT-549 cells. As expected, the accumulation of HIF-1α protein levels depended on the enzymatic activity of PDK1. Mass spectrometry experiments were performed in wild-type BT-549 cells and PDK1 knockout BT-549 cells to identify the phosphorylation modification sites of HIF-1α. We identified a phosphorylation site on S451 of HIF-1α in wild-type BT-549 but not in knockout cells. In addition, previous studies reported that the HIF-1α Ser 451 phosphorylation level was increased in response to hypoxia, and the HIF-1α Ser451 phosphorylation disrupted the interaction between HIF-1α and pVHL/PHD.^26^ pVHL and PHD are involved in the classical HIF-1α degradation pathway, and the weakened interaction between HIF-1α and pVHL/PHD can reduce its ubiquitination level and thus improve its protein stability.^32^^,^^33^ We proposed that PDK1 interacted with HIF-1α and maintained the protein stability through phosphorylation of its serine 451 sites.

Once HIF-1α accumulates in the cytoplasm, it forms a heterodimer with ANRT and enters the nucleus as a transcription factor.^34^^,^^35^ A growing body of evidence shows that HIF-1α forms a complex with p300 and CBP upon nuclear translocation to enhance the transcription of downstream genes, including angiogenic, glycolytic, and anti-apoptotic pathways.^36^, ^37^, ^38^ It has also been reported that tumor suppression can be achieved by disrupting the interaction between HIF-1α and its co-activator p300/CBP.^39^^,^^40^ Our study demonstrated that PDK1 could promote the protein stability of HIF-1α. Combined with the nuclear location of PDK1, we attempted to explore whether nuclear PDK1 could promote the transcriptional activity of HIF-1α.

Quantitative PCR showed that the mRNA levels of the downstream HIF-1α target genes in wild-type BT-549 cells were significantly higher than those in PDK1 knockout BT-549 cells after the two cell groups were treated with MG132 to equalize HIF-1α protein levels. Moreover, PDK1 could enhance the transcription of HIF-1α downstream genes and promote HIF-1α transcriptional activity. Activation of HIF-1α induced angiogenic molecules, such as VEGF, ANGPT1, and EGF. The angiogenesis assay showed that the supernatant from PDK1-expressing tumor cells had a more potent pro-angiogenic effect. Finally, we explored the mechanism by which PDK1 promotes HIF-1α transcriptional activity and found that PDK1 could enhance the binding of HIF-1α to p300. We suggested that PDK1 increased HIF-1α transcriptional activity by enhancing the interaction between HIF-1α and its transcriptional cofactor p300.

## Conclusion

**Figure 7:**
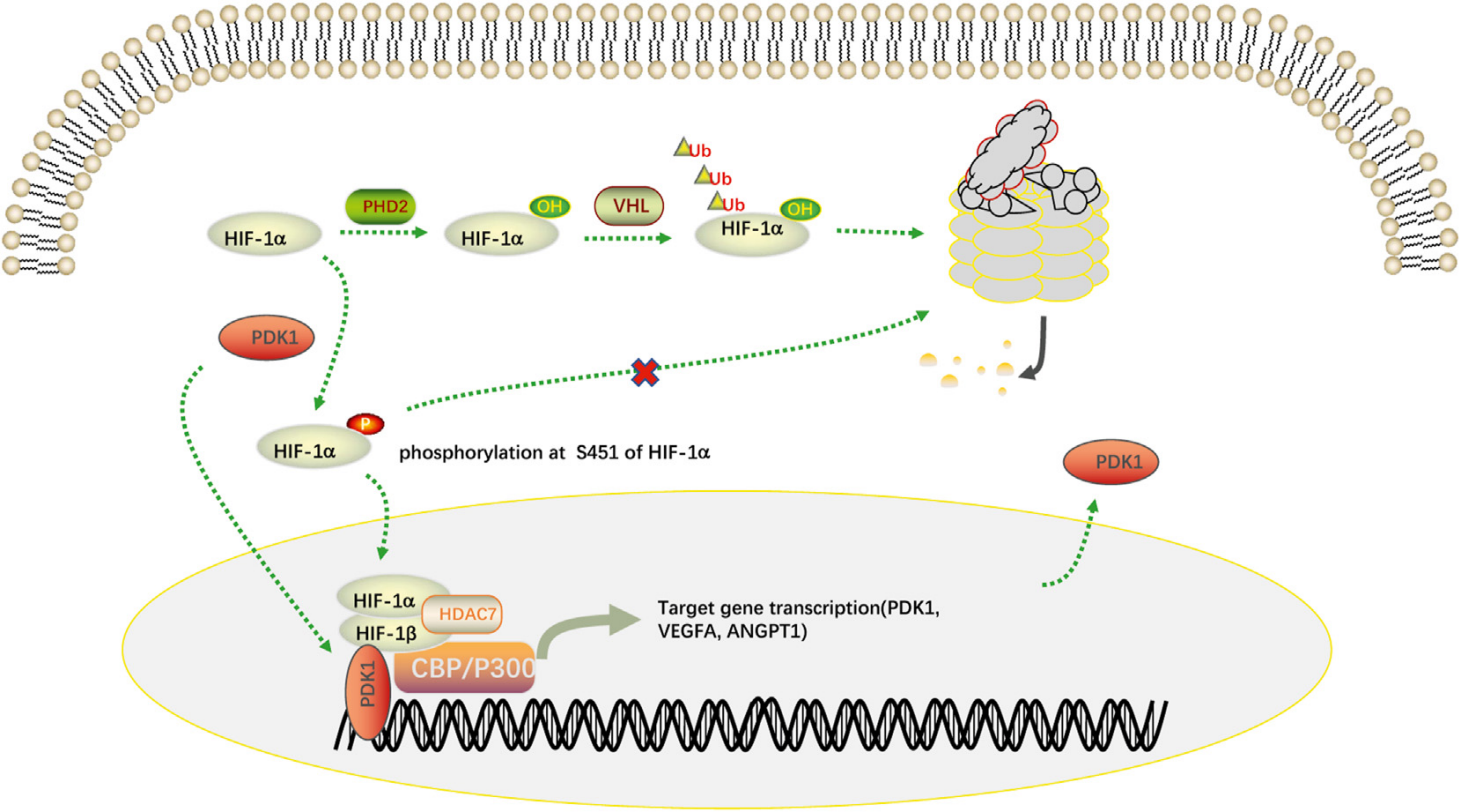
Schematic diagram of the whole research results.

This study demonstrated that PDK1 promoted HIF-1α protein stability by phosphorylating HIF-1a at Ser 451 and enhanced its transcriptional activity by stabilizing the binding of HIF-1α to p300. Concurrently, PDK1, as a classical downstream target gene of HIF-1a, formed a positive feedback loop with HIF-1a to promote the progression of breast cancer (Fig. 7A).

## Conflict of interests

The authors declare no potential conflict of interests.

## Funding

This work was supported by grants from the 10.13039/501100001809National Natural Science Foundation of China (No. 82073255) and the Foundation of Chongqing Municipal Education Commission (China) (No. HZ2021006).
