# Supplementary material for: PDK1 promotes breast cancer progression by enhancing the stability and transcriptional activity of HIF-1α
Source: Genes Dis. 2023 Jul 15;11(4):101041. doi: 10.1016/j.gendis.2023.06.013 (PMC10978537; doi:10.1016/j.gendis.2023.06.013)
Supplement: Multimedia component 1 [file mmc1.docx]

Supporting Information

**Dual oncogenic roles of PDK1 in breast cancer based on the promotion of stability and transcriptional activity of HIF-1α**

*Yu Wei^1^, Dian Zhang^1^, He Shi^1^, Husun Qian^1^, Hongling Chen^1^, Qian Zeng^1^, Fangfang Jin^1^, Yange Wang^1^, Yan Ye^1^, Zuli Ou^1^, Minkang Guo^1^, Bianqin Guo^2^***,* *and Tingmei Chen^1^**


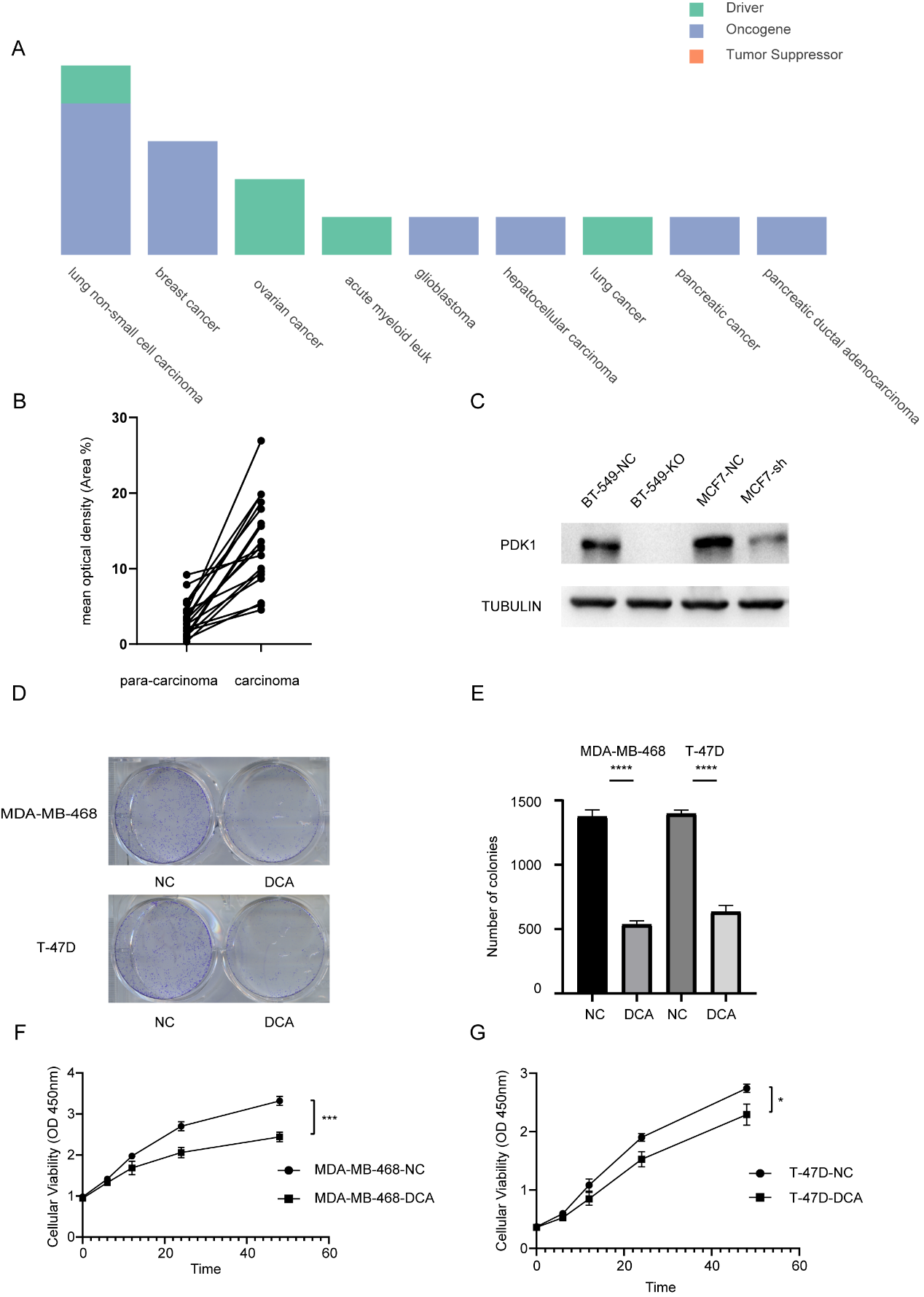


**FigS1. PDK1 was highly expressed in breast cancer. (A)** Oncogenes (promote cancer), tumor suppressors (inhibit carcinogenesis), and drivers (important in cancer development, either oncogene or tumor suppressor) were used as classifiers in the CancerMine database to explore the potential role of PDK1 in the published literature. **(B)** Quantification of immunohistochemistry results for breast cancer tissues and their adjacent tissues (*n*=19). **(C)** The expression level of PDK1 in the indicated cells was detected by western blotting and normalized to TUBULIN as the reference gene. **(D)** Left panel: the clone number of MDA-MB-468 cells and T-47D cells after treatment with DCA for 24 h. Right panel: quantification of colony formation experimental results of three replicates performed under the same condition. **(E)** CCK8 assays were carried out to determine the viability of breast cancer cells after treatment with DCA for 24 h.


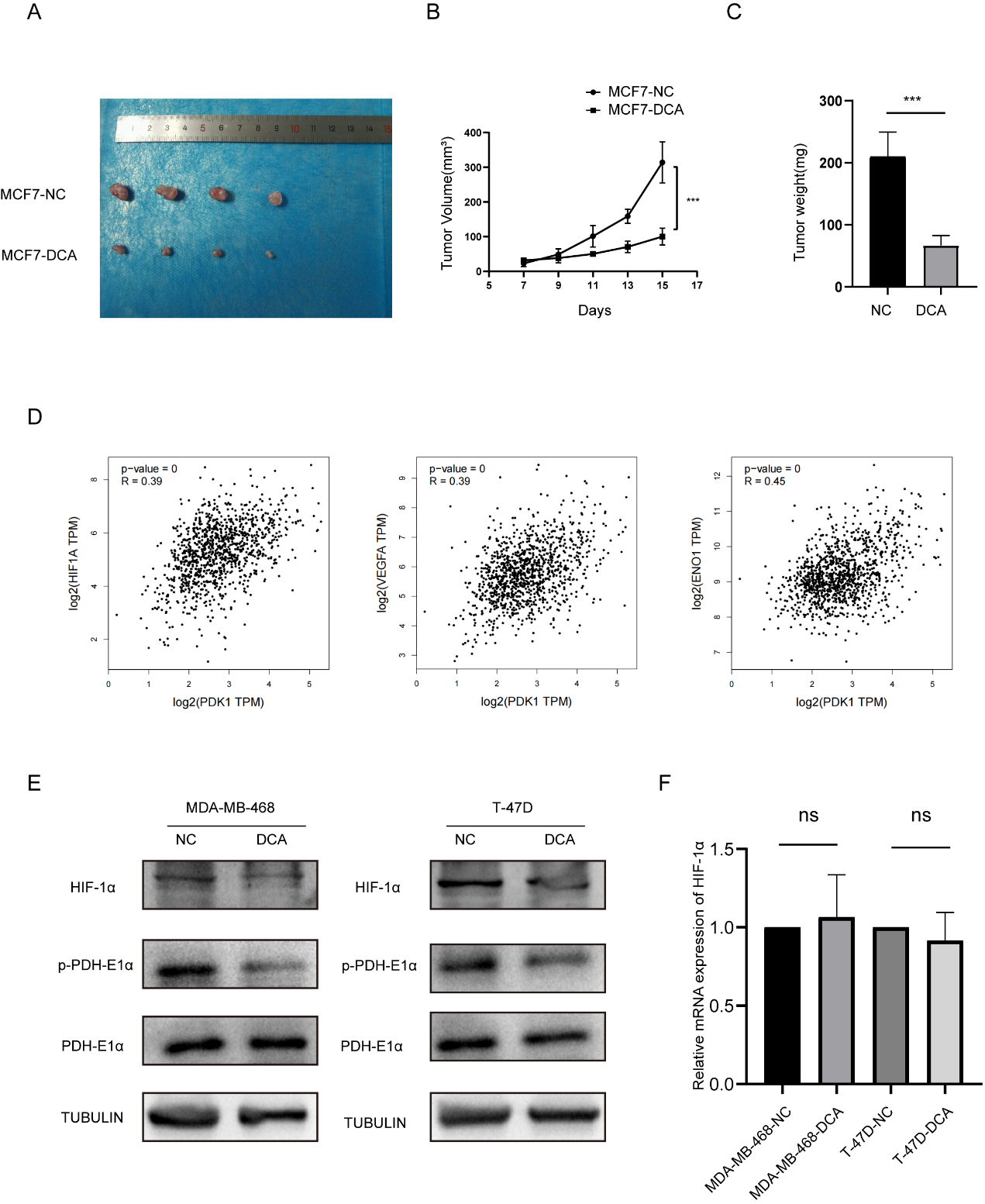


**FigS2. PDK1 promoted tumorigenesis *in vivo* and was positively correlated with HIF-1α downstream genes.** **(A-C)** MCF7 cells were subcutaneously injected into nude mice. After 7 days, PBS (*n*=4) or DCA (1000 mg/kg, *n*=4) was daily intragastric administration for 7 days. Representative size, volume, and weight of tumors from xenograft mice. **(D)** Gene expression correlations were analyzed on the GEPIA platform using the indicated TCGA-provided datasets. (**E**) Expression levels of HIF-1α, p-PDH-E1α, and PDH-E1α in the indicated cells were detected by western blotting and normalized to TUBULIN as the reference protein. Cells were treated with DCA/PBS for 24 h and CoCl_2_ for 6 h before experimentation. (**F**) The mRNA levels of HIF-1α in the indicated cells were measured by qRT-PCR. Gene expression was normalized by TUBULIN (*ns* indicates no significant difference). Cells were treated with DCA/PBS for 24 h and CoCl_2_ for 6 h before experimentation.


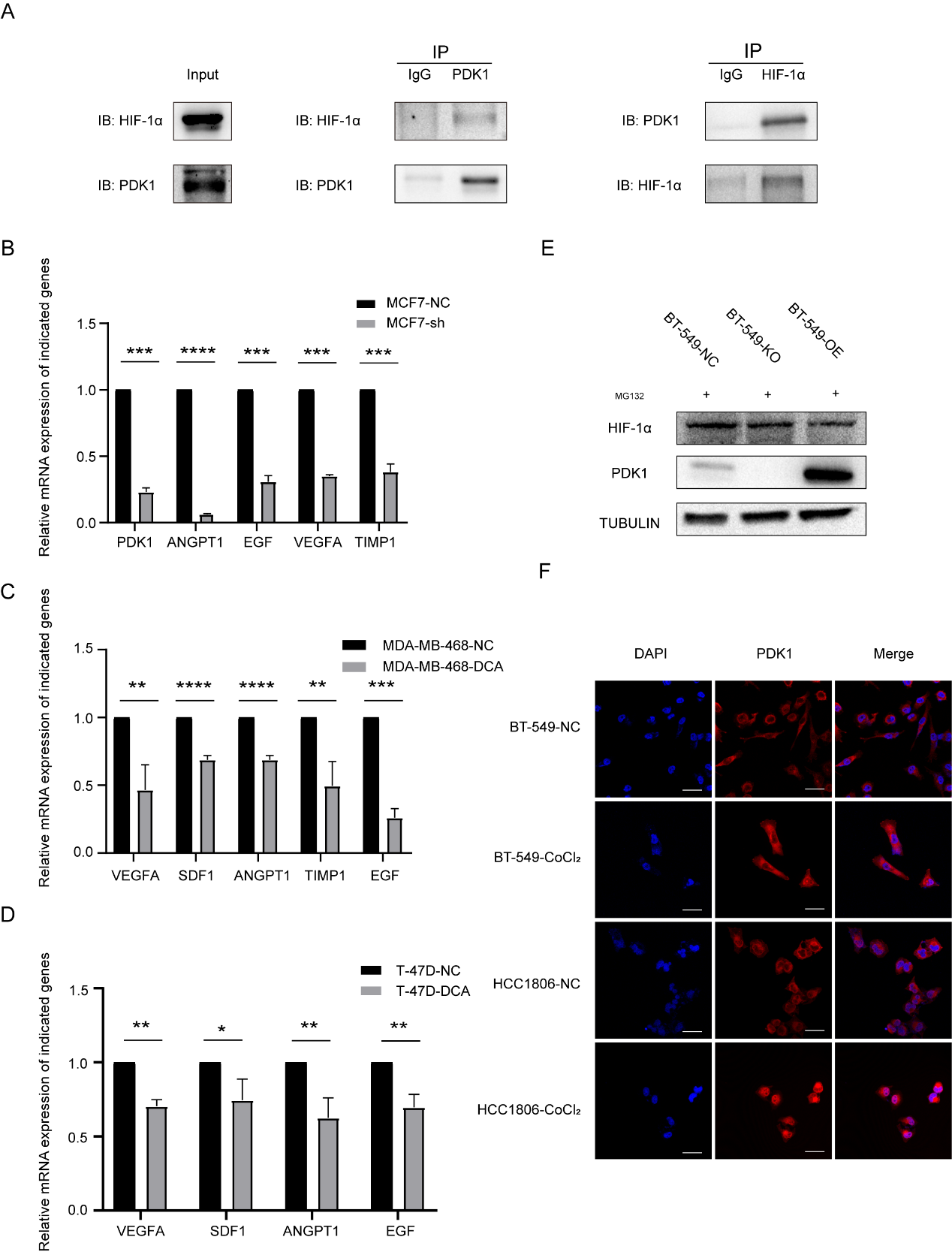


**FigS3. PDK1 interacted with HIF-1α and promoted its transcriptional activity. (A)** Immunoprecipitation assay was performed to detect the interaction between PDK1and HIF-1α in HEK-293T cells. HEK-293T cells that were co-transfected with PDK1 overexpression plasmid and HIF-1α overexpression plasmid and treated with CoCl_2_ for 6 h. **(B)** The mRNA expression of HIF-1α in MCF7-NC and MCF7-sh cells was quantified by Q-PCR. Cells were treated with CoCl_2_ for 6 h. **(C, D)** Q-PCR was performed to detect the gene expression of HIF-1α in the indicated cells. Cells were treated with DCA/PBS for 24 h and CoCl_2_ for 6 h before experimentation. **(E)** Expression level of HIF-1α and PDK1 proteins in the indicated cells was detected by western blotting and normalized to TUBULIN as the reference protein. Cells were treated with DCA/PBS for 24 h and CoCl2 for 6 h and with MG132 for 4 h to balance the HIF-1α expression in different cells. **(F)** Immunofluorescent detection of PDK1 (red) in cells treated with or without CoCl_2_ for 6 h (Scale bars, 50μm).
